# Open-label parallel dose tolerability study of three subcutaneous immunotherapy regimens in house dust mite allergic patients

**DOI:** 10.1186/2045-7022-3-16

**Published:** 2013-05-08

**Authors:** Juliane Rieker-Schwienbacher, Marja J Nell, Zuzana Diamant, Ronald van Ree, Andreas Distler, Johan D Boot, Jörg Kleine-Tebbe

**Affiliations:** 1 Klinikum Stuttgart, Klinik für Dermatologie und Allergologie, Priessnitzweg 24, Stuttgart, Germany; 2 HAL Allergy BV, JH Oortweg 15, Leiden, The Netherlands; 3 Skane University Hospital, Dept. of Respir Med & Allergology, Klinikgatan 18, Lund, S-221 81, Sweden; 4 University Medical Centre Groningen, Dept. of General Practice, P.O. Box 196, Groningen, 9700 AD, The Netherlands; 5 Academic Medical Center (AMC), Department of Experimental Immunology and Department of Otorhinolaryngology, Meibergdreef 9, Amsterdam, The Netherlands; 6 HAL Allergie GmbH, Poststrasse 5, Düsseldorf, Germany; 7 Untersuchungszentrum Dermatologie, Allergologie und Asthma (UZDAA), Spandauer Damm 130 (Haus 9), Berlin, Germany

**Keywords:** Allergic rhinitis, Allergic rhinoconjunctivitis, Conjunctival provocation test, House dust mites, Subcutaneous allergoid immunotherapy, Tolerability, Safety, Short-term effects

## Abstract

**Background:**

The current maintenance dose (10,000 AUeq/monthly) of a subcutaneous allergoid for house dust mite (HDM) immunotherapy has previously shown significant clinical efficacy in patients with HDM induced allergic rhinitis or rhinoconjunctivitis. In order to comply with the 2009 EMA guidelines on immunotherapy products, a study was conducted to evaluate the safety, tolerability and short-term treatment effects of up-dosing regimens with high doses (up to 40,000 AUeq) of allergoid HDM immunotherapy.

**Methods:**

In total 48 patients with HDM-allergic rhinitis or rhinoconjunctivitis (29 M/19 F; 18–53 years) were included and enrolled into one of three up-dosing regimens (1:4:4): 1) a regular regimen with up-dosing to 40,000 AUeq followed by two maintenance doses (total duration 17 weeks), 2) an intermediate regimen (14 weeks) or 3) a fast regimen (11 weeks). Safety and tolerability were evaluated by monitoring of early and late local reactions and systemic reactions. In addition, short-term effects were assessed by conjunctival provocation test (CPT) and levels of serum allergen-specific IgE, IgG and IgG_4_.

**Results:**

Thirty-nine patients completed the study according to protocol. No early local reactions occurred. Late local reactions (LLR) were observed in 12% of the injections. In total, 31 systemic reactions, all grade 1, were reported of which two needed oral antihistamine treatment. No grade 2 or higher systemic reactions were observed. Six patients (15%) did not reach the highest dose due to LLR and/or systemic reactions needing antihistamines (20% in the regular regimen, 16% in the intermediate regimen and 13% in the fast regimen). At the end of the study, an improvement in the CPT was observed in 82.1% of patients, indirectly indicating an early treatment effect at the current dose and higher doses. In addition, IgG_4_ immunoglobulin levels were significantly increased in all groups following treatment.

**Conclusions:**

In this open-label study, allergoid HDM immunotherapy in doses up to 40,000 AUeq was generally well tolerated and no clinically relevant safety issues were identified. In the safety aspects of the three up-dosing regimens no clinically relevant differences were encountered. Therefore, these dose ranges and up-dosing regimens can be safely included in future dose-finding efficacy studies.

## Background

Immunotherapy (IT) with mite extracts is a well-established treatment for patients suffering from allergic rhinoconjunctivitis and/or asthma caused by house dust mite (HDM). Apart from being safe and effective in the treatment of allergic airway disease [1,2], IT has been shown to reduce the development of asthma as well as the onset of new sensitizations in younger patients [3-5].

Traditionally, aqueous extracts have been used for subcutaneous IT. However, soluble proteins can induce severe local and/or systemic allergic adverse reactions upon injection. With the introduction of aluminium hydroxide into allergen vaccines, the proteins are adsorbed to a depot. As a result, allergens are slowly released into the tissue during a longer period of time and thereby reducing the occurrence of adverse reactions [6,7]. Modification of the allergen extracts by glutaraldehyde further reduced the risk of adverse reactions without compromising the immunogenic properties. Several studies have shown that clinical efficacy is retained using modified allergen extracts and that such preparations are considered safer than non-modified allergen extracts [8-10].

The product formulation currently under investigation is a suspension of a glutaraldehyde-modified allergen extract from HDM adsorbed onto aluminium hydroxide. In a previous placebo-controlled study, significant clinical efficacy of one dose strength (10,000 AUeq) of this product was demonstrated in terms of improvements in symptom and medication scores in patients with HDM-allergy after 1 year of treatment [11]. Overall, the product was well tolerated.

According to the current EMA guidelines on the clinical development of IT products [12], dose tolerability studies should be completed before dose-finding efficacy studies can be performed. Accordingly, products should be tested at different doses to provide preliminary data on safety and tolerability with regard to the maximum tolerated dose and a suitable dose escalation scheme. Therefore, a dose tolerability study was conducted using three different up-dosing regimens, all ending at a 4-fold higher dose (40,000 AUeq) compared to the previous study. The primary outcome of the study was the proportion of patients reaching the highest dose. On an exploratory basis, the short-term treatment effects were investigated by the conjunctival provocation test (CPT) and the serum immunoglobulin (Ig) levels.

## Methods

This was a multicentre, open-label, parallel dose, tolerability study with three up-dosing regimens. The study was performed in 6 German centres. All patients were evaluated during a screening visit to assess their baseline condition, including a conjunctival provocation test and baseline serum immunoglobulin levels. Safety and tolerability were evaluated throughout the study. Short-term effects were assessed by CPT and serum Ig levels after patients received 6 doses in the up-dosing regimen and at the end of the study.

### Patients

Patients, aged 18 years or older, suffering from allergic rhinitis or rhinoconjunctivitis related to house dust mites for at least 2 years, with or without concomitant, clinically stable, mild asthma (FEV1 >70% of predicted) were screened.

As verification of the clinical history, sensitizations of all patients were confirmed by a positive (≥ 3 mm mean wheal response) skin prick test (SPT) to HDM (*Dermatophagoides pteronyssinus* (Der p) and/or *Dermatophagoides farinae* (Der f)), a positive CPT to HDM allergen (dose ≤ 10.000 SQ-E/ml), and a positive specific serum IgE test (ssIgE > 0.7 U/ml) for HDM. The main exclusion criteria comprised clinically unstable or more severe asthma (FEV1 ≤70%), any co-sensitization *i.e.*, a positive SPT (≥ 3 mm) to other aero-allergens than HDM if accompanied by clinical symptoms at the time of inclusion. Patients treated with HDM-IT within the last 5 years or other IT at the time of enrolment were excluded from participation. During the study, patients were allowed to use antihistamines or other rescue medication as restrictive as possible. To reduce effects of allergy medication on the study parameters, corticosteroids and antihistamines were not allowed 3 days before the CPT, and antihistamines were not allowed 24 hours before and after IT injection. Eligible patients were sequentially allocated (n=5 / n=20 / n=20) to one of the dose regimens starting with the slowest regimen.

The study protocol (EudraCT number 2008-006261-81) was approved by the relevant Ethics Committees (lead Ethics Committee: LAGeSo, Berlin, Germany) and all patients signed an informed consent form before participation.

### Investigational product and dosing schedules

**Figure 1:**
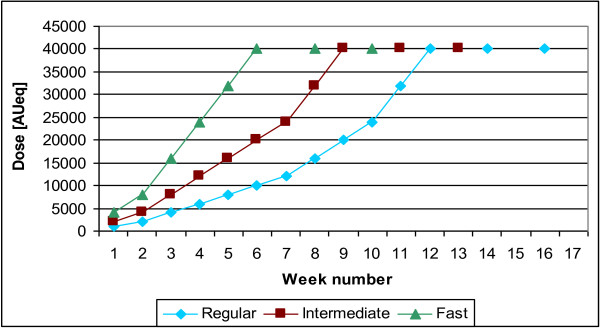
Up-dosing and maintenance phase of the different dosing regimens.

Patients were allocated (1:4:4) to one of three up-dosing regimens (Figure 1) of subcutaneous immunotherapy (SCIT), consisting of a standardized suspension of glutaraldehyde-modified allergoid extract from a mixture of *Dermatophagoides pteronyssinus* and *Dermatophagoides farinae* (1:1) adsorbed onto aluminium hydroxide (PURETHAL® Mites, 20,000 AUeq/ml, HAL Allergy BV, Leiden, The Netherlands, containing major allergen equivalents of 14.0 μg/ml group 1, and 20.0 μg/ml group 2, measured by ELISA in the extract prior to modification and adsorption on aluminium hydroxide).

During up-dosing, SCIT was administered at weekly intervals until the maintenance dose was reached (40,000 AUeq, 2 ml) or no higher dose could be tolerated (see up-dosing rules). The maximum dose was followed by 2 maintenance dose injections with an interval of 2 weeks in all three treatment regimens.

### Up-dosing rules

If the local (early or late) reaction at the injection site was too intense (swelling > 5 cm and ≤ 8 cm), the same dose was repeated. If the swelling was > 8 cm, the next dose was reduced by one step. If that dose was well tolerated, the dose was increased one week later until the maintenance dose was reached. Patients were allowed to receive 4 extra doses to the schedule before reaching the maintenance dose, with a maximum of 2 equal doses in succession. If the patient then still had not reached the intended maintenance dose, the patient was kept on the highest dose reached as the maintenance dose. For mild to moderate systemic reactions (immediate or late) requiring treatment with antihistamines and/or epinephrine, the next dose was reduced by one step in the schedule and the patient was kept on this dose as the maintenance dose.

### Safety and tolerability (clinical evaluations)

Tolerability of the immunotherapy was evaluated by early and late local reactions (*i.e.,* swelling and redness), and systemic reactions after injection. The local reactions were classified into ≤ 5 cm or > 5 cm. As a criterion of tolerability, the maximum number of injections inducing a local swelling of > 5 cm was pre-set on 20% based on expert opinions in daily practice. The systemic reactions were graded into five categories according to Malling *et al.*[13]. Early reactions were recorded by the investigator: *i.e.,* local reactions within 15 minutes after injection and systemic reactions within 30 minutes.

Safety was further evaluated by assessment of vital signs, blood parameters and ECG recording before the start of treatment and at the end of study. If stable, patients were sent home 30 minutes after injection. All patients were instructed to fill out a diary to record late local and systemic reactions and any other adverse event occurring after they had left the clinic.

### Conjunctival Provocation Test (CPT)

A titrated CPT with an aqueous solution of HDM allergen was performed at baseline, at scheduled visit 8 (*i.e.*, one week after reaching 10,000 AUeq, 20,000 AUeq or 40,000 AUeq for the respective dosing regimens), and at the end of study (*i.e.*, two weeks after receiving two maintenance doses, for all regimens). The CPT was performed according Riechelmann et al. [14]. Briefly, the test started with 10 SQ-E/ml (ALK-lyophilisiert HDM SQ Provokationstest, ALK-Scherax, Wedel, Germany) and continued with 100, 1000, 10,000, and 100,000 SQ-E/ml until a positive reaction (itching, redding and discharge) grade II or higher was obtained [15]. To relieve the reaction, patients received a topical antihistamine immediately after completion of the test. The concentrations were prepared on the study site from the stock solution of 100,000 SQ-E/ml on the day of the test. Topical/oral corticosteroids and topical/oral antihistamines were not allowed 3 days before the test.

### Immunoglobulins

Before the start of treatment and at the end of study a blood sample was taken from the patients to quantify specific IgE and specific IgG (IgG and IgG_4_ subtype) to HDM and to the major allergens Der p 1, Der p 2, Der f 1, and Der f 2 by ImmunoCAP analysis. The ImmunoCAP analysis was performed according to instructions of the manufacturer (Thermo Fisher Scientific, Uppsala, Sweden).

### Statistical analysis

Descriptive statistics were performed for all safety and tolerability data. The analysis was performed for all up-dosing regimens combined and for each single regimen separately. Since the main outcome parameter of this study was the safety and tolerability of the up-dosing regimens, compliance to the up-dosing rules of the protocol was essential. Therefore the primary analysis was performed on the per protocol (PP) population.

The analysis of the parameters to assess the short-term treatment effect had an exploratory character. For all these parameters, a Wilcoxon Signed Rank has been applied, if the pre-post-difference in each regimen differed significantly from 0. To test if the pre-post-differences between pairs of regimens differed significantly from each other a Wilcoxon rank-sum-test was used. A significant difference was concluded for all p-values below 0.05. For the analysis of the CPT, the allergen concentrations used were transformed into log CPT values.

## Results

### Population

Forty-eight patients were enrolled in the study and received study medication. Nine patients were excluded from the PP population; 3 patients due to inability to follow the recommended up-dosing regimen, 2 patients due to regular need of antihistamines, 3 patients withdrew their consent and 1 patient was lost to follow-up. Only one patient discontinued the study because of adverse events, occurring after the first 3 doses, all with swellings > 5 cm of mild intensity. This patient was not excluded from the PP population. Characteristics at baseline from enrolled and PP patients are outlined in Table 1.

**Table 1:** Baseline characteristics of safety and per protocol patients per up-dosing regimen

	**Safety population**				**Per protocol**			
	**Regular**	**Intermediate**	**Fast**	**Overall**	**Regular**	**Intermediate**	**Fast**	**Overall**
Patients (n)	6	21	21	48	5	19	15	39
Mean age ± SD (yrs)	27.5 ± 4.9	32.1 ± 11.4	31.2 ± 10.6	31.2 ± 10.4	27.2 ± 5.5	31.8 ± 11.3	33.1 ± 10.4	31.7 ± 10.3
BMI ± SD (kg/m^2^)	27.5 ± 5.9	24.4 ± 3.5	26.7 ± 6.2	25.8 ± 5.2	27.7 ± 6.6	24.5 ± 3.4	26.7 ± 5.9	25.8 ± 4.9
Patients with other allergies n (%)	5 (83.3%)	12 (57.1%)	12 (57.1%)	29 (60.4%)	4 (80%)	10 (52.6%)	9 (60.0%)	23 (59.0%)
SPT HDM *D. pter* ± SD (mm)	6.5 ± 3.1	6.6 ± 2.7	7.4 ± 4.5	6.9 ± 3.6	7.0 ± 3.2	6.6 ± 2.8	8.0 ± 5.2	7.2 ± 3.9
SPT HDM *D. far* ± SD (mm)	7.8 ± 2.6	7.1 ± 1.9	7.0 ± 3.9	7.1 ± 3.0	8.2 ± 2.8	7.0 ± 1.9	7.2 ± 4.4	7.2 ± 3.2
ssIgE ± SD (U/ml)	16.0 ± 19.7	23.4 ± 23.8	26.2 ± 28.2	23.7 ± 25.1	17.1 ± 21.8	24.5 ± 24.7	34.1 ± 29.9	27.3 ± 26.5

### Tolerability

**Figure 2:**
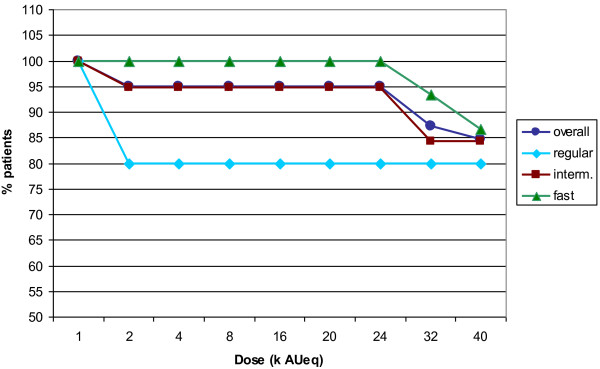
Percentage of patients per regimen reaching a certain dose.

Thirty-nine patients completed the study PP and received 440 subcutaneous injections of HDM immunotherapy. No early local reactions (ELR; swelling > 5 cm) occurred. Late local reactions (LLR; swelling > 5 cm) were observed (see Table 2) in 12% of the injections (7% in the regular regimen, 11% in the intermediate regimen and 16% in the fast regimen). Measurements of skin redness showed similar results (data not shown). In total, 31 systemic reactions (early and late), all grade 1, were reported of which two needed oral antihistamine treatment (see Table 2). In 12 cases presenting with grade 1 reactions also a LLR occurred. No grade 2 or higher systemic reactions were observed. Fifteen percent of patients (6 out of 39, see Figure 2) did not reach the highest dose due to LLR and/or systemic reactions needing antihistamines (20% in the regular regimen, 16% in the intermediate regimen and 13% in the fast regimen). The number and percentage of patients that reached the highest dose are presented in Table 2. The majority of patients (11 out of 15) in the fast dosing regimen had no or only up to two injections giving rise to a LLR before achieving the highest dose.

**Table 2:** Late local and systemic reactions per injection and per patient in each up-dosing regimen and the highest dose reached

**Regimen**	**Total injections (n)**	**LLR n (%) injections**	**LLR n (%) patients**	**SR n (%) injections**	**SR n (%) patients**	**Highest dose reached n (%) patients [95% CI]**	
Regular (n=5)	73	5 (7)	2 (40.0)	4 (5)	4 (80.0)	4 (80.0)	[44.9-100]
Intermediate (n=19)	221	24 (11)	11 (57.9)	23 (10)	9 (47.4)	16 (84.2)	[67.8-100]
Fast (n=15)	146	23 (16)	10 (66.7)	4 (3)	3 (20.0)	13 (86.7)	[69.5-100]
Overall (n=39)	440	52 (12)	23 (59.0)	31 (7)	16 (41.0)	33 (84.6)	[73.3-95.9]

LLR= late local reaction (swelling > 5 cm), SR= systemic reaction (grade 1, early and late), CI=confidence interval.

### Safety

Safety results are presented for the safety population, which consisted of 48 patients that received any study medication. In all three regimens, the majority of the AEs was assessed as at least possibly related to treatment and being of mild intensity (82.8% overall; regular regimen: 69.0%, intermediate regimen: 94.8%, fast regimen: 74.7%). In 17.2% of cases the AE was reported with moderate intensity and no severe AEs were reported. The most commonly reported adverse event was injection site swelling, which occurred in 66.7% of the patients. Other frequently reported related events included nasopharyngitis (33.3%), headache (27.1%), arthralgia and injection site pain (both 14.6%). Most of the adverse events occurred during the up-dosing phase. The pattern of adverse events was similar for all three up-dosing regimens.

No clinically relevant changes in safety laboratory values were observed following treatment except for one patient in the regular up-dosing regimen. This patient had an increased level of ALAT (75 U/l; normal upper range value: 45 U/l) at the end of the study. No clinically relevant deviations in systolic and diastolic blood pressure, heart rate and ECG were observed. No differences were detected for any of these parameters among the three treatment regimens.

A data safety monitoring board (DSMB) evaluated all reported reactions of all immunotherapy injections on a weekly basis. No safety warnings or study adaptations were issued by the DSMB.

### Short-term treatment effects

Short-term treatment effects of HDM immunotherapy were assessed by means of two surrogate efficacy markers: *i.e.,* the CPT and serum levels of allergen specific IgG and IgE.

**Figure 3:**
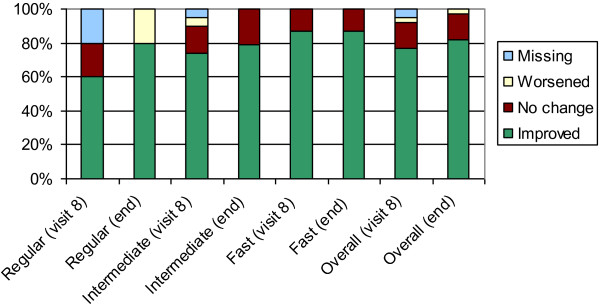
Improvement in conjunctival provocation test (CPT) after 6 doses and at end of study.

The mean lowest concentration that induced a positive (grade II) conjunctival reaction to HDM allergen solution after 6 doses (visit 8) and at the end of study (*i.e*., two weeks after receiving the last maintenance dose) were increased compared to the mean lowest concentration one week before treatment (data not shown). If during a CPT, a patient tolerated a higher dose of the HDM allergen solution after HDM immunotherapy (*i.e.* less sensitive); this was regarded as a sign of efficacy. After 6 doses, 30 out of the 39 PP patients (76.9%, p<0.05) were less sensitive to the CPT. At the end of the study, an improvement was observed in 32 patients (82.1%, p<0.0001). CPT changes in threshold concentration for the 3 up-dosing regimens are shown in Figure 3.

Following treatment, all specific serum IgG_4_ levels were increased in all treatment groups. The different IgG_4_ subclasses showed an increase (p<0.0001) for all up-dosing regimens combined. IgG_4_ was raised by a factor 25.7 for Der p and by a factor 20.9 for Der f. An overview of the specific serum immunoglobulin levels for all regimens combined is given in Table 3. Between the three up-dosing regimens, there was no significant difference in the change in immunoglobulin levels from baseline.

**Table 3:** Specific serum immunoglobulins before and after treatment and the post/pre ratios for the different regimens

		**Regimen**			
**Immunoglobulin**	**Visit**	**Slow**	**Interme-diate**	**Fast**	**Overall**
**IgE Der p (kUA/l)**	Pre-T	13.0 ± 17.6	16.8 ± 18.5	36.0 ± 62.9	23.4±41.1
	Post-T	46.3 ± 77.5	34.4 ± 44.6	53.2 ± 72.2	43.0±59.8
	Ratio	3.6	2.0	1.5	1.8
**IgE Der f (kUA/l)**	Pre-T	15.3 ± 20.9	19.0 ± 21.8	38.1 ± 79.4	25.5 ± 50.9
	Post-T	61.3 ± 108.5	36.5 ± 49.3	56.7 ± 101.2	47.5 ± 79.3
	Ratio	4.0	1.9	1.5	1.9
**IgE nDer p 1 (kUA/l)**	Pre-T	6.0 ± 6.6	7.4 ± 6.4	9.9 ± 10.2	8.1 ± 8.0
	Post-T	21.3 ± 30.1	19.8 ± 17.1	26.2 ± 22.1	22.4 ± 20.6
	Ratio	3.8	2.7	2.6	2.8
**IgE rDer p2 (kUA/l)**	Pre-T	12.1 ± 19.8	14.9 ± 22.3	35.8 ± 72.3	22.2 ± 47.2
	Post-T	28.9 ± 52.8	24.0 ± 33.0	59.1 ± 103.1	38.1 ± 70.8
	Ratio	2.4	1.6	1.7	1.7
**IgG**_**4**_**Der p (μg/l)**	Pre-T	62.1 ± 54.2	128.2 ± 92.5	142.4 ± 176.6	125.6 ± 126.4
	Post-T	1923.0 ± 2656.3	2993.3 ± 3577.2	3961.0 ± 4862.4	3228.3 ± 3997.3
	Ratio	27.8	23.3	27.8	25.7
**IgG**_**4**_**Der f (μg/l)**	Pre-T	67.1 ± 52.2	151.8 ± 161.2	152.1 ± 216.7	140.7 ± 174.0
	Post-T	2445.8 ± 3552.9	2597.3 ±3340.4	3546.2 ± 4954.4	2942.8 ± 3986.7
	Ratio	36.4	17.1	23.3	20.9
**IgG**_**4**_**nDer p1 (μg/l)**	Pre-T	17.3 ± 9.1	51.3 ± 75.1	36.0 ± 25.7	41.2 ± 55.9
	Post-T	739.8 ± 732.3	2128.7 ± 3087.8	1470.9 ± 1406.2	1697.6 ± 2352.7
	Ratio	42.8	41.5	40.9	41.2
**IgG**_**4**_**rDer p2 (μg/l)**	Pre-T	9.2 ± 12.1	45.2 ± 49.8	79.5 ± 139.0	53.1 ± 92.5
	Post-T	1531.0 ± 2126.3	2039.8 ± 2165.6	3295.4 ± 4468.6	2457.5 ± 3245.3
	Ratio	166.4	45.1	41.5	46.3
**IgG nDer p1 (μg/l)**	Pre-T	620.0 ± 389.4	767.4 ±729.4	675.7 ± 384.6	714.2 ± 574.8
	Post-T	2132 ± 1045.7	2835.3 ± 2095.1	2490.0 ± 1048.8	2612.3 ± 1631.2
	Ratio	3.4	3.7	3.7	3.7
**IgG rDer p2 (μg/l)**	Pre-T	674.0 ± 343.1	740.0 ± 529.4	715.7 ± 454.9	722.4 ± 471.4
	Post-T	1986.0 ± 978.3	3241.1 ± 2155.4	4405.3 ± 2699.8	3527.9 ± 2375.9
	Ratio	2.9	4.4	6.2	4.9

nDer = native Dermatophagoides pteronyssinus; rDer = recombinant Dermatophagoides pteronyssinus.

Pre-T = pre-treatment; Post-T = post-treatment; Ratio = Post/Pre-treatment ratio.

## Discussion

*D. pteronyssinus* and *D. farinae* are the most common HDM and are among the most widespread sources of indoor allergens worldwide. These species are very common in humid regions, where most allergic individuals are sensitized to HDM [16].

In this study, the safety and tolerability as well as exploratory short-term effects of three different up-dosing regimens with high doses of allergoid HDM SCIT have been evaluated in patients with allergic rhin(oconjunctiv)itis to HDM. The study had an open-label, parallel-dose, multicentre design. Patients received HDM immunotherapy with a mixture of 50% *Dermatophagoides pteronyssinus* and 50% *Dermatophagoides farinae* (20,000 AUeq/ml) by means of subcutaneous injections according to a regular, an intermediate or a fast regimen, reaching a maximum maintenance dose of 40,000 AUeq.

Fifteen percent of the per protocol patients (6 out of 39) did not reach the maximum dose of 40,000 AUeq due to late local reactions (swelling > 5 cm) or systemic reactions. No early local reactions of > 5 cm occurred and overall 12% of injections gave rise to late local reactions of > 5 cm within 24 hours after injection. These numbers are comparable with the overall numbers of large local reactions found in other SCIT studies with aluminium hydroxide adsorbed HDM extracts [17,18]. The highest percentage (16%) was found in the fast regimen group. However, in none of the up-dosing regimens, the number of injections inducing a local swelling of > 5 cm exceeded 20%, which was pre-set as a criterion of tolerability.

A general risk of immunotherapy with higher allergen extract doses is a potential increase in adverse reactions, particularly systemic ones [19]. In this study, only grade 1 systemic reactions (7% overall) were observed and no systemic reactions of grade 2 or higher. In accordance with a previous study, most systemic reactions occurred below the dose of 10,000 AUeq and did not require treatment [11]. A study by Schubert *et al.*[17] revealed systemic reactions in 4.6% of cases following weekly up-dosing with standardized aluminium hydroxide-adsorbed HDM immunotherapy. These reactions were mostly grade 1–2 and in 0.7% of cases grade 3 [17]. An overview study on side-effects of different allergen-specific SCIT with aluminium hydroxide adsorbed extracts demonstrated that in 1% grade 4 systemic reactions occurred (in 50% of cases a 9.8 μg Der p extract was involved) [19]. Grade 2 or higher systemic reactions are not frequently reported during immunotherapy and therefore, a slight increase in the occurrence of these types of reactions would possibly remain unnoticed in our limited study population. In contrast, substantial increases in systemic reactions would even be observed in small groups (20 patients per arm). Our study did not show any severe reactions, suggesting at least that higher doses of the allergoid HDM immunotherapy are safe and well tolerated. Subsequent studies in larger populations are needed to confirm these findings.

When comparing the local and systemic reactions and the other adverse reactions between the up-dosing regimens, no clear difference in safety and tolerability was observed. Therefore, all up-dosing regimens appeared safe and were well tolerated. Short-term treatment effects were assessed by two surrogate outcome parameters: *i.e.,* the CPT and the serum Ig levels, comparing the pre- *versus* post-treatment changes.

The CPT results at the end of treatment showed an improvement in the threshold concentration in 82.1% of the per protocol patients. Increase of threshold is generally interpreted as a measure of improvement [20]. Improvements were noted in 76.9% of the per protocol treated patients already after approximately 6 doses in all up-dosing regimens. In a previous study with a lower dose of this product (10,000 AUeq) 51% improvement in CPT was observed after 12 months [11]. This previous study was conducted in a similar patient population, but included a placebo group that improved by 30%. The larger number of patients in the current study showing improvement in CPT after shorter treatment may be the result of higher doses of allergoid HDM immunotherapy. However, this needs to be confirmed.

Following treatment, HDM-specific serum Ig levels were increased in all dosing groups compared to pre-treatment levels. These findings indicate that treatment with high doses of allergoid HDM immunotherapy induces an immunological response within a short period of time. Patients who failed to reach the highest maintenance dose had an overall lower immunological response compared to patients reaching the highest dose of 40,000 AUeq. The increase of IgG_4_ Der p levels was higher compared to that found in the previous study using the 10,000 AUeq dose (post-pre ratio of 6.6 in the previous study and 25.7 in the current study) [11]. Similarly, this may be the result of the higher allergoid HDM immunotherapy doses studied, despite a shorter treatment period.

Few studies have examined the effects of mite immunotherapy after approximately 3–4 months of treatment. A study by Ibero *et al*. demonstrated clinical efficacy after 4 months of treatment with a modified HDM extract (cumulative dose 216.75 μg) in asthmatic children by decreases in the allergen-induced airway and skin responses [21]. In our study the cumulative allergoid doses ranged from 346.8 to 467.5 μg, depending on the up-dosing regimen. Overall, the short-term treatment effects in our study indicate an altered immune response following high dose allergoid HDM immunotherapy, also affecting the local conjunctival allergic response. Obviously, these data need confirmation in extended placebo-controlled studies, currently ongoing.

## Conclusions

In conclusion, results of this study showed that updosing towards a maintenance dose of 40,000 AUeq in 6 weekly injections with allergoid HDM immunotherapy was overall safe and well tolerated. In addition, there were early signs of potential treatment effect already after 4 months of treatment. These results warrant future dose-finding efficacy studies.

## Abbreviations

AE: Adverse event; CPT: Conjunctival provocation test; ELR: Early local reaction; DSMB: Data Safety Monitoring Board; HDM: House dust mite; Ig: Immunoglobulin; IT: Immunotherapy; LLR: Late local reactions; PP: Per protocol; SCIT: Subcutaneous immunotherapy; SPT: Skin prick test.

## Competing interest

MJN, DB, and AD are employees of HAL Allergy. ZD and RvR are consultants for HAL Allergy. JRS received remuneration from HAL Allergy for patient inclusion. JKT advised on the design of the study, received remuneration from HAL Allergy for patient inclusion and has received honoraria from HAL Allergy, Germany for educational CME-activities.

## Authors’ contributions

MJN wrote the first version of the manuscript. JKT and MJN contributed to the design of the study. JKT, JRS were involved in collection of the data. RvR was involved in the analysis and interpretation of the immunoglobulin data. ZD, DB, MJN and AD contributed to the interpretation of the data. MJN, DB and ZD contributed to the preparation and critical revision of the manuscript. All authors approved the final version of the manuscript.
